# *Lactobacillus fermentum* 016 Alleviates Mice Colitis by Modulating Oxidative Stress, Gut Microbiota, and Microbial Metabolism

**DOI:** 10.3390/nu17030452

**Published:** 2025-01-26

**Authors:** Huachun Pan, Shumin Yang, Md. F. Kulyar, Hongwei Ma, Kewei Li, Lihong Zhang, Quan Mo, Jiakui Li

**Affiliations:** 1 College of Veterinary Medicine, Huazhong Agricultural University, Wuhan 430070, China; pans@webmail.hzau.edu.cn (H.P.); fakharealam786@hotmail.com (M.F.K.); mahongwei100@126.com (H.M.); lzx591387332@163.com (K.L.); moquan@mail.hzau.edu.cn (Q.M.); 2 National Key Laboratory of Agriculture Microbiology, College of Veterinary Medicine, Huazhong Agricultural University, Wuhan 430070, China; shuminyang@webmail.hzau.edu.cn; 3 College of Animal Science & Technology, Gansu Agricultural University, Lanzhou 730070, China; zhanglih@gsau.edu.cn

**Keywords:** *Lactobacillus fermentum*, ulcerative colitis, gut microbiota homeostasis, microbial metabolism, oxidative stress

## Abstract

Ulcerative colitis (UC) is a chronic and progressive inflammatory gastrointestinal disease closely associated with gut microbiota dysbiosis and metabolic homeostasis disruption. Although targeted microbial therapies are an emerging intervention strategy for inflammatory bowel disease (IBD), the mechanisms by which specific probiotics, such as *Lactobacillus fermentum* 016 (LF), alleviate UC remain unclear. The current study evaluated the effects of LF supplementation on gut health in a basal model using C57BL/6 mice. Subsequently, the preventive effects and mechanisms of LF supplementation on DSS-induced UC were systematically investigated. According to our findings, LF supplementation revealed immunoregulatory capabilities with significantly altered gut the composition of microbiota and metabolic activities, particularly enhancing tryptophan metabolism. In the UC model, LF supplementation effectively mitigated weight loss, increased the disease activity index (DAI), and alleviated diarrhea, rectal bleeding, and colon shortening. Moreover, it reduced colonic pathological damage and histological injury scores. LF intervention improved antioxidant markers and intestinal mucosal barrier function with the activation of the Nrf2–Keap1 signaling pathway and regulation of systemic inflammatory markers, i.e., IL-1β, IL-6, TNF-α, IFN-γ, IL-4, and IL-10. Importantly, LF supplementation reversed metabolic disturbances by significantly increasing the abundance of beneficial genera (e.g., *g_Dubosiella*, *g_Faecalibaculum*, *g_Odoribacter*, *g_Candidatus_saccharimonas*, *g_Roseburia*, and *g_Eubacterium_xylanophilum_group*) and elevating tryptophan metabolites (e.g., melatonin, kynurenic acid, 3-indoleacetic acid, 5-methoxytryptophan, and 5-hydroxyindoleacetic acid). In conclusion, *Lactobacillus fermentum* 016 exhibits potential for regulating gut microbiota homeostasis, enhancing tryptophan metabolism, and alleviating UC, providing critical insights for developing probiotic-based precision therapeutic strategies for IBD.

## 1. Introduction

Inflammatory bowel disease (IBD) is an immune-mediated chronic gastrointestinal ailment defined by repeating periods of relapse and remission. It mostly comprises ulcerative colitis (UC) and Crohn’s disease (CD) [1,2]. The colonic mucosal layer is mostly affected in UC, with chronic lesions. The main manifestations of the disease are diarrhea with rectal bleeding, abdominal pain, and weight loss [3,4]. In recent years, IBD has become increasingly prevalent in developed countries and is rapidly spreading to developing regions, significantly reducing patients’ quality of life and posing a major global public health challenge [5,6]. The pathogenesis of IBD is highly complex, involving factors such as environmental influences, genetic susceptibility, gut microbiota dysbiosis, pathogenic infections, and aberrant immune responses [7,8]. Current therapeutic strategies for IBD commonly include aminosalicylates, corticosteroids, and immunomodulators. However, these treatments are limited in efficacy and often associated with significant adverse effects [9]. Consequently, researchers are actively exploring more effective and safer treatment approaches, such as microbial therapy [10,11], fecal microbiota transplantation (FMT) [12,13], anti-inflammatory dietary interventions [14], plant extract-based interventions [15,16], stem cell therapy [17], and biologic therapy [18]. Among these, microbial therapies, as a natural, safe, and effective intervention strategy, have emerged as a promising research hotspot, garnering significant attention from the scientific community.

Probiotics are a class of live microorganisms beneficial to host health, and their inclusion in diets has been extensively studied, demonstrating their ability to promote gut health [19,20]. The gut microbiota, often referred to as the “hidden organ”, play a pivotal role in maintaining host physiological functions [21]. Members of the *Lactobacillus* genus, as dominant commensals in the gastrointestinal tract, are widely involved in immune regulation and the maintenance of gastrointestinal homeostasis. Cheng et al. reported that *Lactobacillus gasseri* JM1 alleviates intestinal inflammatory damage in UC mice by modulating intestinal tight junction proteins and cytokine expression [22]. Additionally, supplementation with *Lactobacillus gasseri* NK109 inhibited NF-κB activation and restored the balance of gut microbiota, thereby reducing systemic inflammation and alleviating cognitive impairment [23]. Gu et al. revealed that *Lactobacillus plantarum* ZJ316 attenuated UC exacerbation by improving the many helpful bacteria and reducing harmful genera, thus rebalancing inflammatory cytokine levels [24]. Moreover, other studies have revealed that *Bacteroides fragilis* can mitigate intestinal injury in necrotizing enterocolitis (NEC) by suppressing the FXR-NLRP3 signaling pathway, thereby restoring gut microbiota dysbiosis and bile acid metabolism disorders [25]. In summary, probiotics have been widely studied and proven to be effective in improving gut health, including reducing intestinal inflammation, regulating gut–brain interactions, and ameliorating specific gut-related diseases. However, the efficacy of different strains varies significantly, and individual responses to probiotics are influenced by factors such as genetics, diet composition, and baseline gut microbiota [26]. Therefore, when using probiotic-based therapies to intervene in gut-related diseases, it is crucial to consider these factors to achieve more precise and effective therapeutic outcomes.

Tryptophan metabolism, as a critical physiological process in the body, plays essential roles in immune regulation, nervous system health, gut homeostasis, and various chronic diseases [27,28]. Recent studies have highlighted the significant therapeutic potential of tryptophan (Trp) and its metabolites in treating intestinal disorders [29,30]. Indeed, it was found that the severity of intestinal inflammation inversely correlates with xanthurenic acid (XANA) and kynurenic acid (KYNA) levels. Both metabolites modulate intestinal epithelial cells and T-cell functions, reducing colitis severity. In addition, recombinant aminoadipate aminotransferase was reported to modulate endogenous tryptophan metabolism and colitis [31]. Gut microbiota have recently gained attention as a key player in tryptophan metabolic pathways for host health. Jiang et al. showed that transplantation of Trp-enriched gut microbiota promoted the abundance of *Lactobacillus* and *Parabacteroides*, two genera of beneficial bacteria, which enhanced indole metabolite production, activated 5-HT receptor 2B (HTR2B), and largely improved the experimental colitis condition [32]. Tryptophan metabolites, as key mediators of gut microbiota–host interactions, play vital roles in maintaining gut homeostasis. For example, intervention with *Lactobacillus plantarum* AR495 restored serotonin (5-HT) in the colon, thereby regulating gastrointestinal motility and inflammatory responses and alleviating diarrhea symptoms in irritable bowel syndrome (IBS) [33]. Melatonin, a crucial product of the tryptophan metabolic pathway, is central to microbial metabolism, circadian rhythms, and gut mucosal immune cell interactions, providing new targets for the treatment of intestinal diseases [34,35]. Recent research by Jia et al. showed that melatonin pretreatment effectively alleviated colistin-induced intestinal inflammation and mucosal injury. It was helpful to maintain the abundance of useful bacteria, such as *Bifidobacteriales* and *Erysipelotrichales*, while repressing the proliferation of pathogenic bacteria such as *Desulfovibrionales*, hence regulating gut microbiota balance [36]. Besides, tryptophan metabolites are involved in many critical biological functions and inflammation or disease modulation, such as indole-3-lactic acid and indole-3-acetic acid. Li et al. demonstrated that ILA derived from *Bifidobacterium breve* promotes AKT phosphorylation, inhibits M1 macrophage polarization, alleviates colitis, and suppresses tumorigenesis [37]. Furthermore, Zhang et al. found that ILA produced by *Lactobacillus plantarum* L168 enhances the dendritic cell secretion of IL-12, thereby activating CD8^+^ T-cell immune function and improving colorectal tumorigenesis and colitis [38]. In conclusion, these studies underscore the significant clinical potential of strategies targeting tryptophan metabolism for treating intestinal diseases.

The imbalance of intestinal homeostasis, such as the disturbance of intestinal flora and metabolic homeostasis, is closely related to the occurrence and development of UC [39,40]. Consequently, developing interventions capable of effectively modulating gut homeostasis to prevent and treat UC has become a research hotspot. Our previous studies have demonstrated that *Lactobacillus fermentum* 016 (LF) possesses excellent acid and bile salt tolerance, as well as antioxidant properties. Moreover, LF can effectively impede the growth of different pathogenic bacteria, including *Escherichia coli*, *Staphylococcus aureus*, *Salmonella typhimurium*, and *Pasteurella multocida* [41]. Additionally, LF exhibits strong adhesion to intestinal epithelial cells, suggesting its potential for UC prevention. The purpose of this current study is to systematically evaluate the consequences of LF supplementation on gut health in C57 mice and further investigate the protective role and molecular mechanisms in DSS-induced ulcerative colitis. We first discuss, under normal physiological conditions, the possible effects of LF supplementation on gut health, microbial structure, and metabolism in C57 mice. Subsequently, we explore the prophylactic effects of LF supplementation on UC mitigation and its underlying mechanisms. The underlying mechanisms may involve improvements in antioxidant capacity, improvements in intestinal barrier function, reductions in inflammatory responses, and modulation of the structure and metabolic action of gut microbiota. This work could provide the theoretical foundation for LF as a novel probiotic strain in UC prevention and treatment and open up a new therapeutic avenue for intestinal diseases.

## 2. Materials and Methods

### 2.1. Chemicals and Reagents

Dextran sulfate sodium (DSS, M.W. 36–50 kDa) was procured from MP Biomedicals (Santa Ana, CA, USA). Sigma Chemical Co. (Darmstadt, Germany) supplied sulfasalazine (SASP). MRS medium was acquired from Qingdao Hope Bio-Technology Co., Ltd. (Qingdao, China). T-SOD, GSH-Px, CAT, MDA, and MPO kits were acquired from Nanjing Jiancheng Bioengineering Institute Co., Ltd. (Nanjing, China). Jianglai Biotechnology Co., Ltd. (Shanghai, China) provided ELISA kits for IL-1β, IL-6, TNF-α, IFN-γ, IL-4, and IL-10. Abclonal Technology Co., Ltd. (Wuhan, China) provided antibodies for β-actin, ZO-1, occludin, claudin-1, E-cadherin, Keap1, Nrf2, HO-1, NOQ-1, and HRP. The other compounds used in the experiment were analytically pure and obtained commercially.

### 2.2. Strains and Culture Conditions

The *Lactobacillus fermentum* 016 strain used in this study was previously isolated by our research group from the gastrointestinal tract of healthy yaks in the Tibetan region of China. LF was cultured anaerobically in an MRS medium at 37 °C for approximately 12 h. The bacterial suspension was then centrifuged to remove the supernatant, and the bacterial bead was rinsed with saline solution (0.9% Nacl). At the end, the bacterial concentration was adjusted to approximately 1.0 × 10^9^ CFU/mL for subsequent experimental applications.

### 2.3. Animals

This research used 7-week-old male C57BL/6J strain mice, each weighing 22 ± 2 g (*n* = 72), sourced from the Laboratory Animal Center of Huazhong Agricultural University (Wuhan, China). Animals were reared under SPF conditions in a setting with a temperature of 22 ± 2 °C and relative humidity of 50 ± 5%, using a 12 h light/dark cycle. All mice had unrestricted access to regular laboratory mouse feed and drinking water at all times. Mice were acclimatized for 7 days to the experimental settings to mitigate any stress associated with handling prior to the commencement of formal trials. All experimental protocols adhered to the guidelines established by the Animal Care and Welfare Committee of Huazhong Agricultural University (HZAUMO-2024-0231) and the pertinent regulations outlined in the Hubei Province Laboratory Animal Management Regulations.

### 2.4. Animal Experimental Design

A double-blinded experimental design was conducted to explore the effects of LF supplementation on gut health and DSS-induced colitis of C57BL/6J mice. The mice were randomly divided into six groups, a number of 12 mice each, and subjected to two experimental settings:

Experiment I consisted of the Control and LF groups. The Control group was given 100 μL of saline solution (0.9% Nacl) daily by gavage; the mice in the LF group were orally administered the same volume of 1.0 × 10^9^ CFU/mL LF bacterial suspension every day. The entire experiment lasted for 21 days.

Experiment II consisted of the Control, DSS, SASP + DSS, and LF + DSS groups. The DSS group received 2.5% DSS in drinking water ad libitum to induce ulcerative colitis. The SASP + DSS group was treated with 200 mg/kg sulfasalazine (SASP) via oral gavage alongside DSS-induced colitis [42]. The LF + DSS group was supplemented with 1.0 × 10^9^ CFU/mL of LF via oral gavage during DSS induction. The experimental period for Experiment II was 28 days. All treatments were conducted strictly according to the experimental protocol, and relevant data were collected for subsequent analyses.

### 2.5. Disease Activity Index (DAI) and Colon Length

The DAI model was used to determine the severity of UC in all groups of mice. Throughout the DSS therapy period, body weight, stool consistency, and the intensity of diarrhea and rectal bleeding were monitored and documented daily. All calculations for DAI score were made by averaging the results for body weight change, stool constancy, and fecal occult blood. Finally, at the end of the experiment, each group of mice were euthanized. The abdominal skin was cut with scissors to expose the entire abdominal cavity. The spleen was removed, weighed, and recorded. Subsequently, the entire colon was carefully removed, rinsed with normal saline, and the length was measured and then photographed. Finally, the colon tissue and contents were collected into a freezing bottle, quickly cooled in liquid nitrogen, and stored in an ultra-low temperature refrigerator at −80 °C.

### 2.6. Histological Analysis of Colon Tissue

Approximately 0.5 cm of tissue was taken from the center of the colon, rinsed with normal saline to eliminate any contaminants from the lumen, and then preserved in 4% paraformaldehyde for 24 h. The tissues were then fixed in paraffin. The paraffin blocks were sectioned into 5 μm thick slices, deparaffinized with xylene, and rehydrated using a gradually increasing ethanol concentration. Hematoxylin and eosin staining were performed in accordance with our laboratory’s regular protocols. Then, the sections of tissues were imaged by an optical microscope, and the degree of pathological damage was evaluated according to the crypt scoring system: 0, normal crypt structure; 1, 1/3 loss of the crypt base; 2, 2/3 loss of the crypt base; 3, complete loss of the crypt, but the surface epithelium is still intact; 4, complete loss of crypts and surface epithelium [43].

### 2.7. Detection of Oxidative Stress-Related Physiological Biochemical Indicators and MPO in Colon Tissue

The serum was collected from the mouse, and the content of MDA, GSH-Px, T-SOD, and CAT was assayed by following the manufacturer’s instructions for the corresponding assay kits. The kits were received from Nanjing Jianchen Bioengineering Institute in Nanjing, China. The colon tissue samples were carefully weighed and homogenized in an ice bath with PBS buffer (*w*/*v*, 1:9) using a handheld tissue homogenizer (Tissue Ruptor II, QIAGEN) at 12,000 rpm for 45 s. The homogenate was centrifuged for 10 min at 4 °C at 5000× *g* to obtain the supernatant. MPO activity was measured using a myeloperoxidase assay kit in accordance with the manufacturer’s procedure. The MPO assay kit was obtained from Nanjing Jianchen Bioengineering Institute in Nanjing, China.

### 2.8. Measurement of Serum Inflammatory Cytokines

The levels of cytokines were assessed using the appropriate enzyme-linked immunosorbent assay (ELISA) kits. The technique was carried out exactly as described in the manufacturer’s instructions included with the kits. A variety of cytokines were analyzed: interleukin-1β, interleukin-6, tumor necrosis factor-α, interferon-γ, interleukin-4, and interleukin-10.

### 2.9. Immunohistochemical and Immunofluorescence Assays

The paraffin sections were deparaffinized in xylene before being hydrated using a graded ethanol series. Microwave antigen retrieval was conducted for 20 min in 0.01 M citrate buffer (pH 6.0). To suppress endogenous peroxidase activity, slides were cooled to room temperature and incubated in 3% H_2_O_2_. Slides were blocked with blocking buffer at room temperature for 1 h to prevent nonspecific staining. Slides were then treated overnight at 4 °C with a primary antibody: ZO-1 and occludin. Immunostaining was carried out by using the ABC peroxidase method (Vector Laboratories) and DAB was used as the chromogenic substrate and hematoxylin for counterstaining. For immunofluorescent staining, paraffin sections were made for antigen retrieval in the same 0.01 M citrate buffer, pH 6.0, by microwave treatment. The sections were treated with primary antibodies such as ZO-1, claudin-1, and E-cadherin, followed by secondary antibodies from Thermo Fisher Scientific, 1:400. Finally, the sections were counterstained with DAPI in mounting medium.

### 2.10. Western Blot Analysis

The colon tissues were mixed with RIPA lysis buffer (Beyotime Biotechnology, China) and a protease inhibitor (Roche, Basel, Switzerland), then homogenized on a homogenizer (T10 basic, IKA), and protein concentrations were determined using a BCA Protein Assay Kit (Beyotime Biotechnology, Haimen, China). Following denaturation, 30 µg of each protein was separated using 10% SDS-PAGE and transferred to a PVDF membrane (GE Healthcare). The membrane surface was blocked with 5% non-fat milk and then incubated with primary antibodies against β-actin, occludin, claudin-1, Keap1, Nrf2, HO-1, and NOQ-1 (ABclonal, Wuhan, China) overnight at 4 °C. The membranes were then treated with the integrating secondary antibody for 1 h at 25 °C. Protein expressions were observed using an enhanced chemiluminescence (ECL) reagent (LifeiLab, AP34L015, Shanghai, China).

### 2.11. Extraction of Bacterial Genomic DNA and 16S rDNA Sequencing Analysis

Fecal samples were collected and kept in sterile freezing tubes. The microbial genomic DNA from colon contents was extracted using the QIAamp DNA Stool Mini Kit (Qiagen, Hilden, Germany). Ultimately, DNA quality and integrity were evaluated using 1% agarose gel electrophoresis and spectrophotometric analysis at 260/280 nm. The 16S rRNA V3–V4 variable region was amplified using universal primers 338F (5′-ACTCCTACGGGGGGCAG-3′) and 806R (5′-GACTACHVGGGTWTCTAAT-3′). The PCR products were then purified with the AxyPrep DNA Gel Extraction Kit from Axygen Biosciences in California, USA, in accordance with the manufacturer’s instructions. The sequencing libraries were created using the NEXTFLEX Rapid DNA-Seq Kit. Sequencing was conducted using the Illumina MiSeq platform. Fastq (version 0.20.0) conducted quality-based filtering of raw readings, whereas FLASH (version 1.2.7) combined reads with adapters. The purified and optimized sequences were then categorized into OTUs using UPARSE (version 7.1) with a 97% sequence similarity threshold. The RDP classifier (version 2.2; confidence threshold, 0.7) was used to categorize the representative sequences of each OTU. The Ace, Chao, Shannon, and Simpson indices were used to measure alpha diversity. Beta diversity was evaluated via the Bray–Curtis distance and then displayed by principal component analysis (PCA). LefSe analysis was used to identify microbial taxa that were differentially represented across groups.

### 2.12. Non-Targeted Metabolomics Analysis of Colonic Contents

Metabolites were extracted using 400 µL of a methanol–water solution (4:1, *v*/*v*) after the precise weighing of 50 mg of colon material. Following precipitation at −20 °C, the samples were treated using a high-throughput tissue grinder (Wonbio-96c, Shanghai Wanbo Biotechnology Co., Ltd., Shanghai, China) at 50 Hz for 6 min, then vortexed for 30 s and subjected to ultrasonic treatment at 40 kHz for 30 min at 5 °C. The samples were then frozen at −20 °C for 30 min to assist protein precipitation. The supernatant was aliquoted into sample vials after centrifuging at 13,000× *g* for 15 min at 4 °C. The raw UPLCMS/MS data were pre-processed with Progenesis QI v2.3 software from Waters, Milford, USA. The sample sequence was randomized to remove systematic variation. Metabolites were identified using a number of reliable databases, such as Shanghai Meiji Biopharmaceutical Technology Co., Ltd.’s self-built database, HMDB http://www.hmdb.ca/ (accessed on 15 April 2024) and Metlin https://metlin.scripps.edu/ (accessed on 15 April 2024). Following database searches, data were moved to the Meiji Bio Cloud platform (https://cloud.majorbio.com (accessed on 20 April 2024)) for integrated analysis, which included matrix file differential analysis and data preparation. To conduct PLS-DA and PCA analysis, the R language ropls package (Version 1.6.2) was utilized. Significant differential metabolites were assessed using the criteria VIP ≥ 1 and *p* < 0.05 based on fold change analysis and Student’s *t*-test. The KEGG database (KEGG, https://www.kegg.jp/kegg/pathway.html (accessed on 1 May 2024)) was then used to map the metabolic pathways associated with the substantially different metabolites. To uncover the possible biological processes, metabolic pathway enrichment analysis was carried out.

### 2.13. Statistical Analysis

The experimental data were presented as the mean ± SEM, analyzed using GraphPad Prism 8.3.0 (GraphPad Software, San Diego, CA, USA) and the Excel program (Microsoft, Redmond, Washington, USA). A two-tailed unpaired Student’s *t*-test was used in order to determine the differences among the groups. One-way analysis of variance (ANOVA) with Tukey’s multiple comparisons was used for the multiple comparisons. The statistical significance criteria were set at * *p* < 0.05, ** *p* < 0.01, and *** *p* < 0.001.

## 3. Results

### 3.1. Effect of LF Supplementation in the Diet on Gut Health and Immune Cytokines in C57 Mice

**Figure 1:**
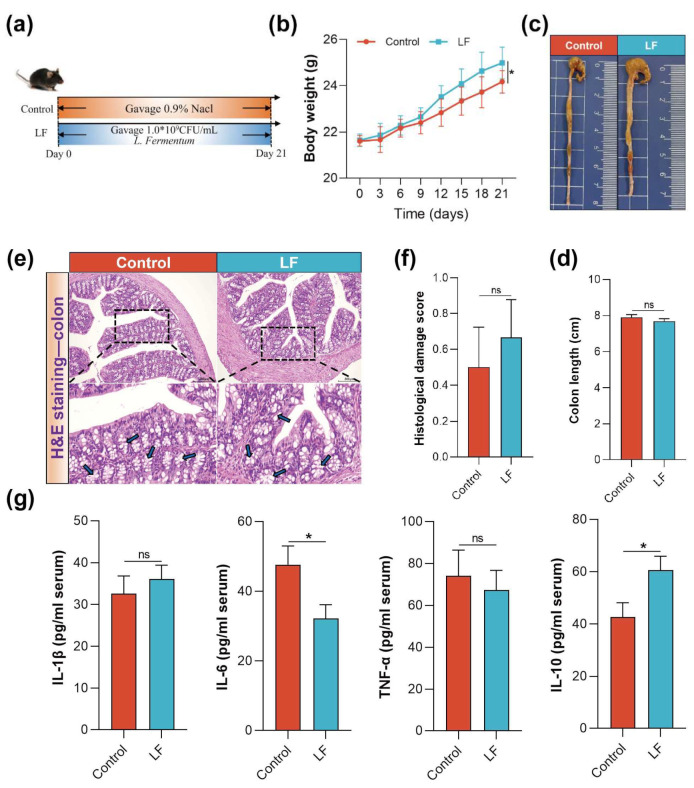
Modulatory effects of LF supplementation in the diet on gut health and immune factors in C57 mice. (**a**) Schematic plan of the experimental design. (**b**) Body weight change curve of mice during the experiment. (**c**,**d**) Measurement and statistical analysis of colon length. (**e**,**f**) Histopathological sections of colon tissue and tissue damage scores (scale bar: 200 μm). (**g**) ELISA detection of serum cytokine levels. Data are presented as mean ± SEM. ns statistically insignificant, * *p* < 0.05, ns non-significant.

To evaluate the impact of LF supplementation in the diet on gut health in C57 mice, we designed a 3-week animal experiment (Figure 1a). The results showed that LF supplementation had no adverse effects on the growth, development, or overall health of the C57 mice. Mice in the LF group exhibited normal weight gain trends throughout the experimental period, similar to the Control group (*p* > 0.05, Figure 1b), and maintained a good mental state and normal coat color throughout the study. Colon length is one of the key indicators of overall colon health, and no significant difference in colon length was observed between the LF and Control groups (*p* > 0.05) (Figure 1c,d). Histopathological analysis further confirmed the safety of LF supplementation, with observations showing that the colon crypt structure was intact in the LF group, with no inflammatory cell infiltration, abundant goblet cells, or abnormal tissue morphology. Additionally, the tissue damage score showed no significant differences (Figure 1e,f). Importantly, serum inflammatory cytokine analysis revealed that LF exhibited some immune-modulating effects. While the levels of pro-inflammatory cytokines, i.e., IL-1β and TNF-α remained stable (*p* > 0.05), the level of IL-6 was significantly reduced (*p* < 0.05), and the content of the anti-inflammatory cytokine IL-10 was notably increased (*p* < 0.05) (Figure 1g).

### 3.2. Effects of LF Supplementation in the Diet on Gut Microbiota and Microbial Metabolic Processes in C57 Mice

**Figure 2:**
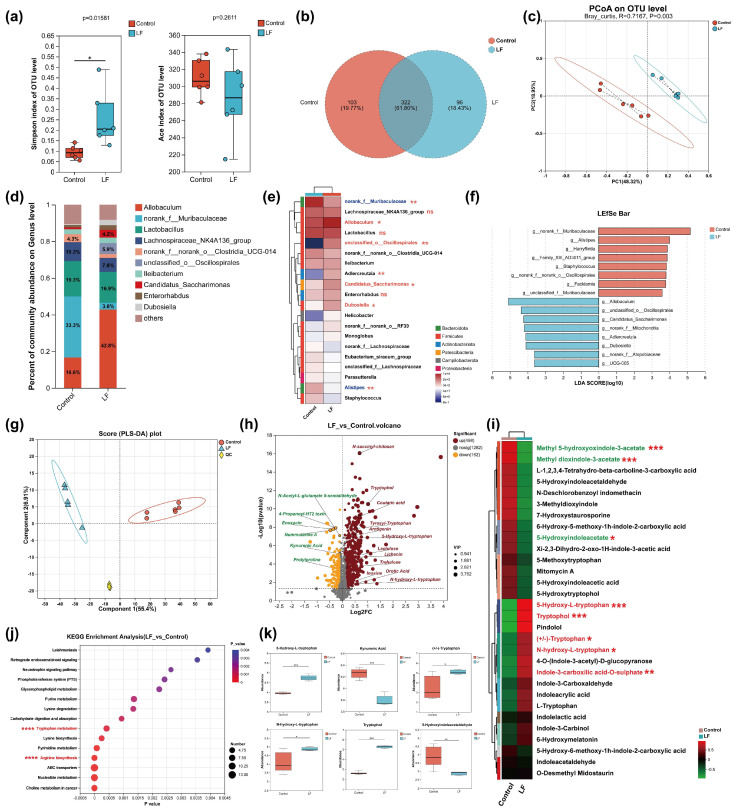
Effects of LF supplementation in the diet on the colonic microbiota and microbial metabolism in C57 mice. (**a**) α-diversity index analysis, including Simpson index and Ace index. (**b**) Venn diagram analysis. (**c**) β-diversity analysis, displayed by PCoA. (**d**) Community bar plot analysis (at the genus level). (**e**) Community heatmap analysis (at the genus level). (**f**) LefSe analysis showing microbial taxonomic differences. (**g**) Partial least squares discriminant analysis (PLS-DA). (**h**) Volcano plot analysis of differential metabolites. (**i**) Heatmap analysis of differential metabolites (averaged values). (**j**) KEGG pathway enrichment analysis. (**k**) Bar chart of inter-group metabolite differences. Data are presented as mean ± SEM. * *p* < 0.05, ** *p* < 0.01, *** *p* < 0.001, **** *p* < 0.0001, ns non-significant.

To elucidate the regulatory effects of LF supplementation on the gut microbiome and its metabolism in C57 mice, we conducted a comprehensive analysis of the gut microbiota and metabolomic profiles after LF intervention. Alpha diversity analysis showed that LF supplementation reduced the diversity of the microbiota (Simpson index, *p* < 0.05) but did not alter the microbial richness (Ace index, *p* > 0.05) (Figure 2a). Venn diagram analysis revealed that both groups shared 322 operational taxonomic units (OTUs), accounting for 61.8%, while LF supplementation specifically enriched 96 OTUs, representing 18.43%, indicating that LF intervention induced a selective enrichment response of certain OTUs (Figure 2b). β-diversity analysis of the gut microbiota, assessed by principal coordinates analysis (PCoA), showed distinct clustering patterns in the LF group, further confirming that LF supplementation significantly reshaped the composition of the mouse gut microbiome (Figure 2c). At the genus level, we observed that LF supplementation caused significant changes in the abundance of several functional bacterial genera. Specifically, LF supplementation led to a significant increase in the abundance of beneficial genera such as *g_Allobaculum*, *g_Candidatus_saccharimonas*, *g_Adlercreutzia*, *g_Dubosiella*, and *g_unclassified_o_Oscillospirales*. In contrast, the Control group showed higher abundances of genera such as *g_Alistipes*, *g_Harryflintia*, and *g_Staphylococcus* (Figure 2d–f). Notably, *Staphylococcus* is a potential pathogenic genus that poses a threat to host health.

Subsequently, we utilized non-targeted metabolomics based on high-resolution liquid chromatography–mass spectrometry (LC–MS) to reveal the impact of LF on colonic microbial metabolism. Partial least squares discriminant analysis (PLS-DA) demonstrated significant differences in the metabolic profiles between the LF and Control groups (Figure 2g). Volcano plot and heatmap analyses of the differential metabolites showed that continuous LF supplementation led to the upregulation of 498 metabolites and downregulation of 162 metabolites. Notably, we observed significant enrichment of key intermediate metabolites in several tryptophan metabolic pathways in the LF group, including 5-hydroxyl-L-tryptophan, (+/−)-tryptophan, tryptophol, kynurenic acid, N-hydroxy-L-tryptophan, 5-hydroxyindoleacetaldehyde, and indole-3-carboxylic acid-O-sulphate (Figure 2h,i,k). KEGG pathway enrichment analysis further confirmed that LF intervention significantly enriched the tryptophan metabolism and arginine biosynthesis pathways (Figure 2j).

### 3.3. Effects of Preventive LF Supplementation on the Development of DSS-Induced Colitis in Mice

**Figure 3:**
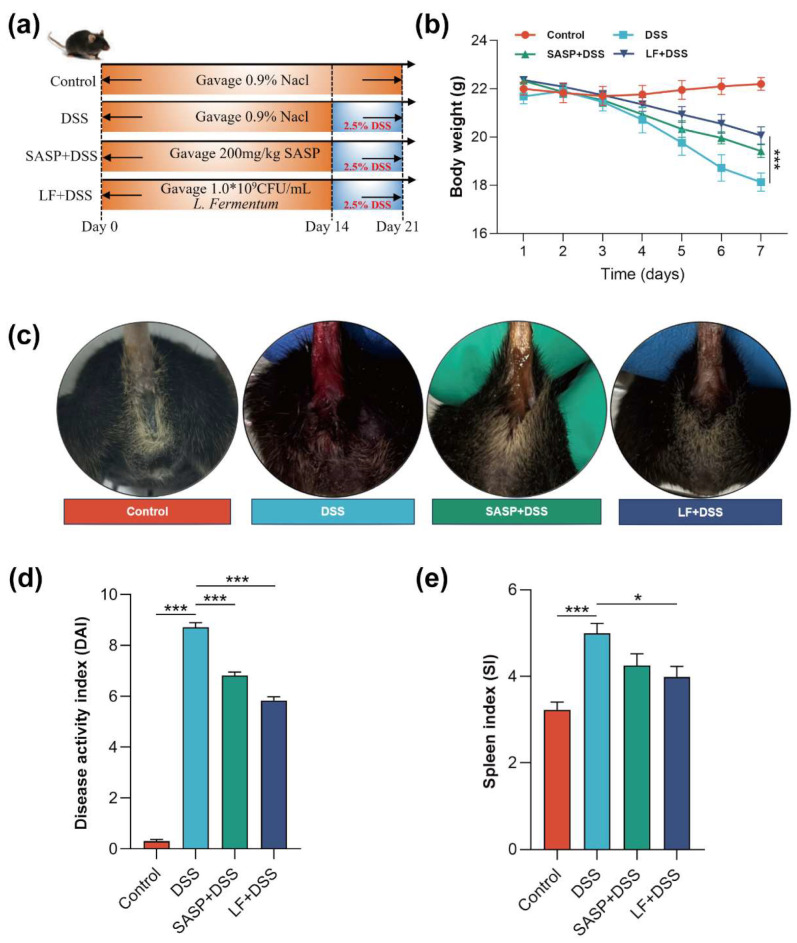
The ameliorative effects of preventive LF supplementation on DSS-induced colitis symptoms. (**a**) Experimental design schematic. (**b**) Body weight change curve of mice during DSS induction. (**c**) Phenotypes of diarrhea and rectal bleeding in mice. (**d**) Disease activity index (DAI). (**e**) Spleen index analysis. Data are expressed as mean ± SEM. * *p* < 0.05, *** *p* < 0.001.

Dextran sulfate sodium (DSS) induces chemical damage to intestinal epithelial cells and disrupts barrier function, effectively simulating the main pathological features of UC [44]. To systematically evaluate the protective effect of preventive LF supplementation on intestinal function, we established a DSS-induced colitis model. As shown in Figure 3a, the experimental design included four groups: the Control group, DSS group, SASP + DSS positive Control group, and LF + DSS group. All mice were fed standard chow (CD). The results demonstrated that preventive LF supplementation significantly alleviated the symptoms of DSS-induced colitis. Compared to the DSS group, preventive LF supplementation significantly reduced body weight loss (*p* < 0.001) (Figure 3b). Moreover, severe diarrhea and rectal bleeding were observed in the DSS group, while preventive LF supplementation and SASP notably improved these symptoms (Figure 3c) and significantly reduced the disease activity index (DAI) (*p* < 0.001) (Figure 3d). Notably, DSS exposure caused a significant increase in spleen index (*p* < 0.001), indicating systemic inflammation activation, while LF intervention significantly improved this (*p* < 0.05) (Figure 3e).

### 3.4. Effect of Preventive LF Supplementation on Colonic Injury in Colitis Mice

**Figure 4:**
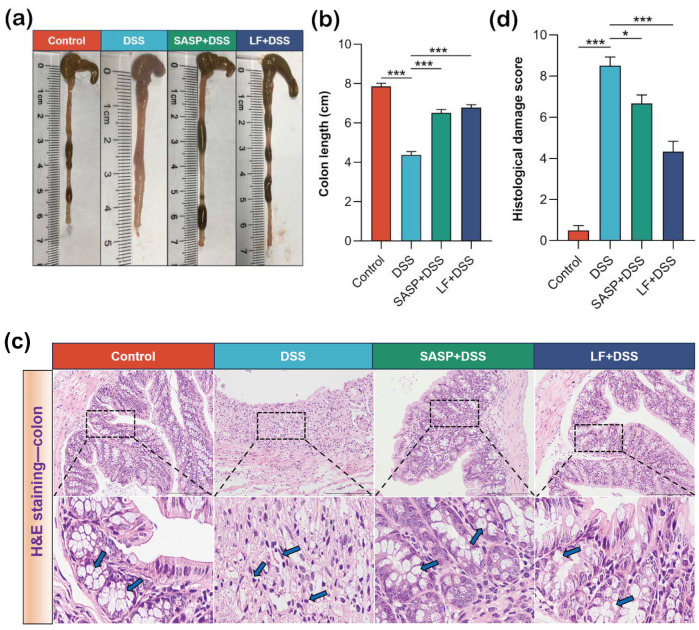
Effects of preventive supplementation of LF on colonic damage. (**a**) Colonic length images. (**b**) Bar graph analysis of colonic length. (**c**) Histological sections of colonic tissue. (**d**) Histopathological damage scores. Data are presented as mean ± SEM. * *p* < 0.05, *** *p* < 0.001.

To assess the protective effect of preventive LF supplementation on DSS-induced colonic tissue damage, we performed histological and pathological analyses. The results showed that DSS treatment significantly shortened the colon length (*p* < 0.001), which is a typical manifestation of colitis. Notably, preventive LF supplementation significantly improved the DSS-induced colon shortening effect (*p* < 0.001) (Figure 4a,b). Further histopathological analysis revealed a series of typical inflammatory changes in the colons of DSS-treated mice, including mucosal edema, destruction of crypt structures, degeneration of epithelial cells, extensive infiltration of inflammatory cells, and goblet cell reduction. These pathological changes were alleviated in the LF + DSS group, showing a relatively intact tissue structure and significantly reduced inflammatory cell infiltration, while the number of goblet cells increased (Figure 4c). More intuitively, the tissue damage score confirmed that preventive LF supplementation significantly reduced the DSS-induced colonic tissue damage score (*p* < 0.001) (Figure 4d).

### 3.5. Effect of Preventive LF Supplementation on Oxidative Stress, Immune Cytokines, and MPO in Colitis Mice

**Figure 5:**
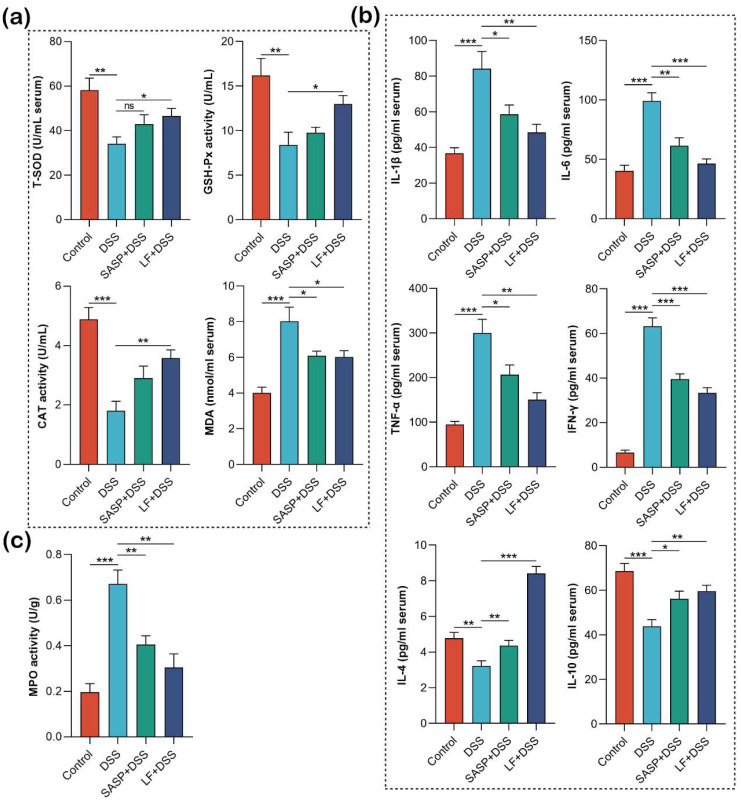
Effects of preventive supplementation of LF on oxidative stress, immune cytokines, and MPO. (**a**) Analysis of oxidative stress-related physiological and biochemical indicators. (**b**) Serum immune cytokine analysis. (**c**) MPO activity in colonic tissue. Data are presented as mean ± SEM. * *p* < 0.05, ** *p* < 0.01, *** *p* < 0.001, ns non-significant.

To elucidate the regulatory effects of preventive LF supplementation on DSS-induced oxidative stress and inflammatory responses, we systematically evaluated changes in relevant biomarkers. First, we assessed the levels of oxidative stress markers. The results revealed that, compared with the Control group, the DSS-treated group exhibited a substantial increase in malondialdehyde (MDA) levels (*p* < 0.001), while the activities of total superoxide dismutase (T-SOD), glutathione peroxidase (GSH-Px) (*p* < 0.01), and catalase (CAT) (*p* < 0.001) were significantly decreased. These phenomena were significantly improved in the preventive LF supplementation group, as indicated by a significant decrease in MDA levels (*p* < 0.05) and a significant increase in antioxidant enzyme activities of T-SOD, GSH-Px (*p* < 0.05), and CAT (*p* < 0.01) (Figure 5a). Next, we measured serum cytokine levels by ELISA (Figure 5b) and found that DSS treatment significantly increased the levels of pro-inflammatory cytokines IL-1β, IL-6, TNF-α, and IFN-γ (*p* < 0.001) in the mouse serum. At the same time, the levels of anti-inflammatory cytokines IL-4 (*p* < 0.01) and IL-10 (*p* < 0.001) were significantly decreased in the DSS group. Notably, compared with the DSS group, the LF + DSS group showed a significant reduction in pro-inflammatory cytokines IL-1β and TNF-α (*p* < 0.01), and a highly significant reduction in IFN-γ and IL-6 (*p* < 0.001). These results suggest that preventive LF supplementation effectively regulates the body’s inflammatory response by decreasing pro-inflammatory cytokines and increasing anti-inflammatory cytokines, thereby balancing the inflammatory state of the body. Finally, we assessed myeloperoxidase (MPO) activity, a key marker of neutrophil infiltration. The results revealed that, compared with the Control group, DSS treatment significantly increased MPO activity in colon tissues (*p* < 0.001) (Figure 5c), indicating substantial neutrophil infiltration at the inflammation site, which exacerbates intestinal inflammation. However, preventive LF supplementation significantly reduced the DSS-induced increase in MPO activity (*p* < 0.01), suggesting that preventive LF supplementation effectively inhibits tissue infiltration by inflammatory cells, alleviates the inflammatory severity of UC, and protects normal intestinal function.

### 3.6. Effect of Preventive LF Supplementation on Intestinal Barrier Function and Nrf2–Keap1 Pathway in Colitis Mice

**Figure 6:**
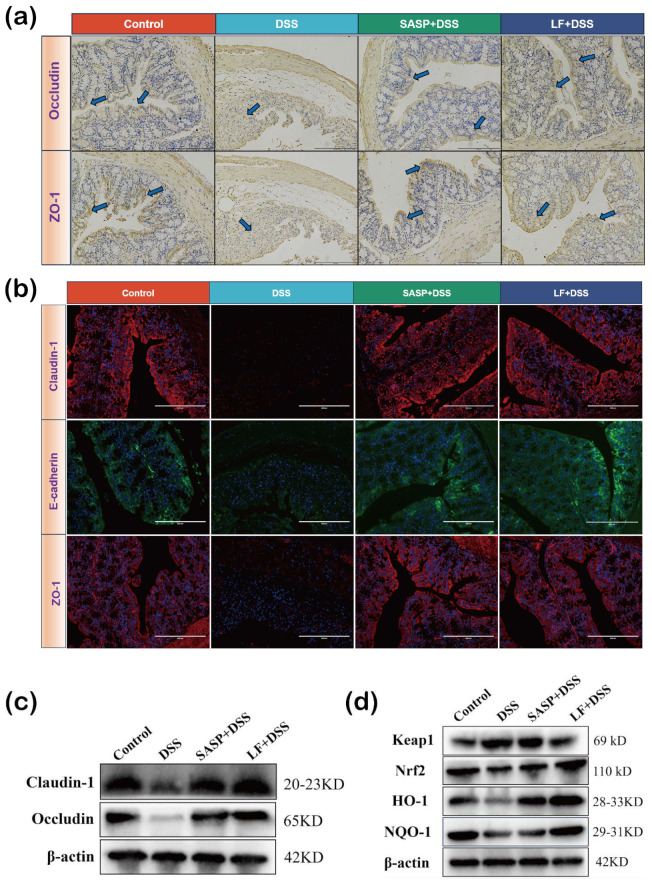
Effects of preventive supplementation of LF on intestinal mucosal barrier function and the Nrf2–Keap1 pathway. (**a**) Immunohistochemistry (IHC) analysis of colonic tissue. (**b**) Immunofluorescence (IFC) analysis of colonic tissue. (**c**) Western blot analysis of intestinal tight junction proteins. (**d**) Western blot analysis of the Nrf2–Keap1 pathway. Data are presented as mean ± SEM. The blue arrows indicate the expression of tight junction proteins in colon epithelial cells.

Tight junction proteins (TJ) are essential for maintaining the integrity of intestinal epithelial cells. To evaluate the restorative effect of preventive LF supplementation on the intestinal epithelial mechanical barrier, we conducted immunohistochemical analysis of TJ protein expression in the colon. The results showed that the colonic expression of ZO-1 and occludin was significantly lower in colitis mice compared to the Control group, whereas these changes were markedly improved after LF intervention, manifested as the expression of ZO-1 and occludin in the colon of LF + DSS group, with SASP + DSS group being similar to the Control group (Figure 6a). Further, immunofluorescence analysis revealed that LF supplementation increased the expression of claudin-1, E-cadherin, and ZO-1 in colon tissues, effectively counteracting the decrease in TJ protein expression caused by DSS exposure, and maintaining the integrity of the intestinal epithelial barrier (Figure 6b).

To further elucidate the molecular mechanisms by which LF regulates UC, we assessed the activation status of the key oxidative stress response pathway, Nrf2–Keap1. Western blot results (Figure 6c,d showed that, compared to the Control group, DSS exposure upregulated the expression of Keap1 (*p* < 0.05), while inhibiting the expression of the transcription factor Nrf2 and its downstream antioxidant proteins HO-1 and NQO-1 (*p* < 0.05). This dysregulation of the signaling pathway may result in decreased antioxidant defense capacity and exacerbated inflammatory damage. Notably, preventive LF supplementation significantly reduced Keap1 expression (*p* < 0.05), while increasing the expression of Nrf2 and its downstream proteins HO-1 and NQO-1 (*p* < 0.05). These results suggest that preventive LF supplementation enhances the body’s antioxidant and anti-inflammatory capabilities by regulating the Nrf2–Keap1 signaling pathway, maintaining intestinal barrier integrity, and mitigating inflammatory damage.

### 3.7. Effect of Preventive LF Supplementation on the Colonic Microbiota of Colitis Mice

**Figure 7:**
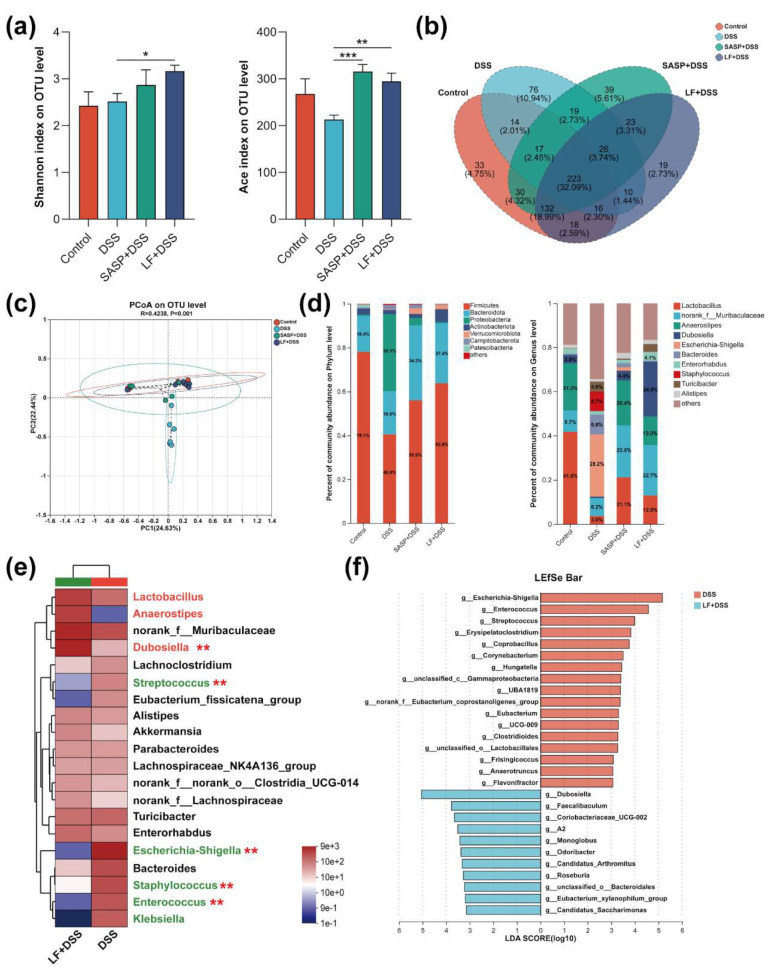
Effects of preventive supplementation of LF on the gut microbiota structure and composition in colitis mice. (**a**) α-diversity index analysis, including Shannon index and Ace index. (**b**) Venn diagram showing the overlap of microbiota among different treatment groups. (**c**) β-diversity analysis, displaying group differences in microbiota through PCoA. (**d**) Bar plots of microbiota composition at the phylum and genus levels. (**e**) Cluster heatmap of microbiota communities at the genus level. (**f**) LefSe analysis showing microbial taxonomic differences. Data are presented as mean ± SEM. * *p* < 0.05, ** *p* < 0.01, *** *p* < 0.001.

To investigate the regulatory effect of preventive LF supplementation on the intestinal microbiota in a DSS-induced UC model, we analyzed the colonic microbiota composition. α-diversity analysis revealed that, compared to the DSS group, LF intervention significantly increased the Shannon index (*p* < 0.05) and Ace index (*p* < 0.01) (Figure 7a), indicating that LF supplementation notably enhanced the diversity and richness of the colonic microbiota in mice. Venn diagram analysis (Figure 7b) showed that the four treatment groups shared 223 OTUs (32.09%) with the DSS group and LF pre-treatment group, having 76 (10.94%) and 19 (2.73%) specific OTUs, respectively. Principal coordinates analysis (PCoA) further revealed that the microbiota communities of the Control and LF pre-treatment groups exhibited significant clustering, whereas the DSS group displayed a more dispersed distribution (Figure 7c), suggesting that LF pre-treatment helps maintain microbiota stability.

As shown in Figure 7d, at the phylum level, the microbiota composition of the three groups, excluding the DSS group, was similar. DSS treatment led to a decrease in the relative abundance of Firmicutes to 40.6%, while the relative abundance of Proteobacteria increased to 35.1%. Notably, LF pre-treatment was able to restore the proportions of Firmicutes (63.8%) and Bacteroidetes (27.4%). At the genus level, DSS treatment increased the relative abundance of several potential pathogenic genera, including *g_Escherichia-Shigella* (28.2%), *g_Staphylococcus* (8.7%), *g_Turicibacter* (4.6%), and others, while decreasing the proportion of beneficial genera such as *g_Lactobacillus* (3.6%) and *g_norank_of_muribaculaceae* (8.2%). However, following LF intervention, these trends were reversed, with increased proportions of *g_Lactobacillus* (12.9%), *g_norank_of_muribaculaceae* (22.7%), *g_Anaerostipes* (13.0%), and *g_Dubosiella* (24.9%) compared to the DSS group.

Furthermore, we focused on genus-level differential analysis. Community distribution heatmaps and LDA discriminant analysis (Figure 7e,f) revealed that LF pre-treatment significantly increased the abundance of several beneficial genera, including *g_Dubosiella, g_Faecalibaculum, g_Candidatus_saccharimonas, g_Coriobacteriaceae_UCG-002, g_Monoglobus, g_Odoribacter, g_Roseburia*, and *g_Eubacterium_xylanophilum_group*. In contrast, DSS treatment significantly increased the abundance of several opportunistic pathogenic genera, including *g_Escherichia-Shigella, g_Enterococcus, g_Streptococcus, g_Staphylococcus, g_Klebsiella, g_Erysipelatoclostridium, g_Coprobacillus*, and *g_Corynebacterium*. These results suggest that preventive LF supplementation can significantly modulate the composition of intestinal microbiota, increase the abundance of beneficial genera, decrease the abundance of pathogenic genera, balance gut homeostasis, and promote intestinal health.

### 3.8. Effects of Preventive LF Supplementation on Colonic Microbial Metabolic Processes in Mice with Colitis

**Figure 8:**
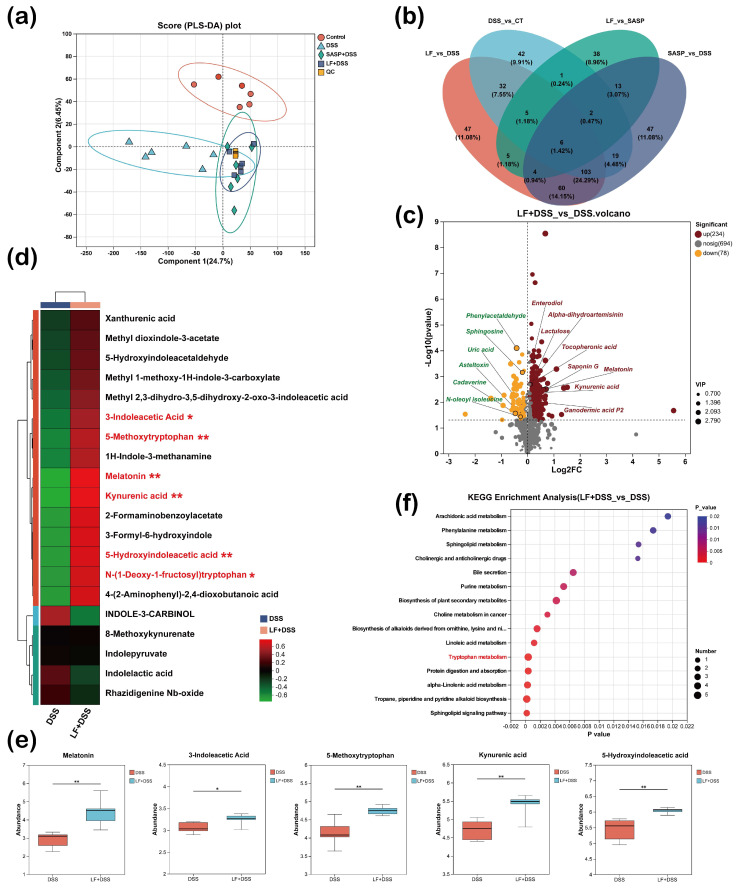
Effects of preventive supplementation of LF on the gut microbiota metabolite profile in colitis mice. (**a**) Partial least squares discriminant analysis (PLS-DA) showing metabolite differences between groups. (**b**) Venn diagram illustrating the overlap of metabolites among different treatment groups. (**c**) Volcano plot highlighting significantly altered metabolites. (**d**) Heatmap analysis of metabolites in the tryptophan metabolism pathway. (**e**) Bar chart of metabolite differences between groups. (**f**) KEGG pathway enrichment analysis of metabolites. Data are presented as mean ± SEM. * *p* < 0.05, ** *p* < 0.01.

To elucidate the regulatory effects of preventive LF supplementation on colonic microbial metabolism in UC mice, we performed an untargeted metabolomic analysis of colonic contents. Partial least squares discriminant analysis (PLS-DA) (Figure 8a) revealed distinct metabolic profiles between the DSS group and the other groups, indicating substantial differences in metabolite composition. Venn diagram analysis further demonstrated (Figure 8b) that compared to the DSS group, the LF intervention group exhibited 47 significantly altered metabolites (11.08%), which may serve as key mediators of LF’s protective effects. Volcano plot analysis of metabolites (Figure 8c) revealed that preventive LF supplementation significantly altered 312 metabolites compared to the DSS group, with 234 metabolites upregulated and 78 downregulated. Notably, several metabolites with unique biological functions were significantly upregulated, including melatonin, kynurenic acid, enterodiol, alpha-dihydroartemisinin, lactulose, tocopheronic acid, saponin G, and ganodermic acid P2. Conversely, some potentially harmful metabolites, such as phenylacetaldehyde, sphingosine, uric acid, asteltoxin, cadaverine, and N-oleoyl isoleucine, were significantly downregulated.

Further heatmap analysis of metabolites related to tryptophan metabolism and pathways involving indole and its derivatives revealed that LF pretreatment significantly increased levels of various tryptophan metabolic products, notably melatonin, kynurenic acid, 5-methoxytryptophan, and 5-hydroxyindoleacetic acid (*p* < 0.01). Additionally, 3-indoleacetic acid and N-(1-deoxy-1-fructosyl)tryptophan exhibited a significant upward trend (*p* < 0.05) (Figure 8d,e). KEGG pathway enrichment analysis showed that preventive LF supplementation notably influenced multiple metabolic pathways, most of which were closely related to energy and nutrient metabolism, such as the sphingolipid signaling pathway, linoleic acid metabolism, and amino acid metabolism (Figure 8f). Notably, the tryptophan metabolism pathway was significantly enriched, with the most pronounced increases observed in melatonin and kynurenic acid levels. These findings systematically demonstrate for the first time that preventive LF supplementation effectively modulates gut microbial metabolism, increasing levels of beneficial metabolites, reducing levels of potentially harmful metabolites, and enhancing the tryptophan metabolism pathway, thereby alleviating colitis.

## 4. Discussion

With the evolving environment and lifestyle changes, the global incidence of inflammatory bowel disease (IBD) continues to rise, making it a significant public health challenge that threatens human health [6,45]. In recent years, microbial therapies, particularly probiotics, have shown tremendous potential in IBD treatment due to their multifunctional regulatory capabilities [46,47]. Specifically, probiotics exert their therapeutic effects through multiple mechanisms, including strengthening the intestinal mucosal barrier, optimizing the composition of gut microbiota, modulating immune responses, and enhancing metabolic activities [48,49]. A healthy mucosal barrier is critical for maintaining intestinal homeostasis and defending against external harmful substances. Probiotics enhance this barrier function through various molecular mechanisms. For example, Wu et al. found that *Limosilactobacillus reuteri* strengthens intestinal tight junctions and suppresses inflammation by promoting *Bacteroides acidifaciens* to produce pentadecanoic acid, thereby inhibiting NF-κB activation [50]. Additionally, probiotics regulate the composition and proportion of microbial populations, restoring the diversity of gut microbiota. Liu et al. demonstrated that *Lactobacillus plantarum* CCFM8610 effectively alleviated diarrhea-predominant irritable bowel syndrome (IBS-D) by reducing the abundance of *Methanobrevibacter* and increasing butyrate-producing bacteria [51]. In terms of immune modulation, probiotics maintain immune homeostasis and prevent excessive immune responses that can damage intestinal tissues. For instance, *Lactobacillus acidophilus* and its metabolite ursodeoxycholic acid (UDCA) alleviate UC symptoms by activating the RapGap/PI3K-AKT/NF-κB signaling pathway, regulating Treg cells and M1 macrophages [52]. Similarly, *Lactobacillus johnsonii* alleviates colonic inflammation by producing indole-3-lactic acid (ILA), which activates the aryl hydrocarbon receptor (AhR) [53]. This study aims to systematically evaluate the protective effects of LF supplementation on ulcerative colitis (UC), focusing on its regulation of intestinal mucosal damage, barrier integrity, microbial community composition, and metabolic homeostasis. By uncovering the potential mechanisms of LF’s protective effects, this research provides new theoretical insights and candidate strains for the application of probiotics in the prevention and treatment of IBD.

Probiotics, as critical modulators of gut health, have been demonstrated to balance the intestinal microbiota, enhance gut barrier function, and regulate host immune responses [54]. This study initially assessed the impact of LF supplementation on gut health in C57 mice. The results revealed that LF supplementation alone reduced IL-6 secretion while increasing IL-10 levels, thereby improving the balance of inflammatory cytokines and exhibiting potential anti-inflammatory effects. Microbiota analysis demonstrated that LF supplementation significantly altered the composition of the microbiota, particularly by increasing the abundance of beneficial genera, such as *g_Allobaculum*, *g_Candidatus saccharimonas*, *g_Adlercreutzia*, *g_Dubosiella*, and *g_unclassified_o_Oscillospirales*. Metabolomic analysis further revealed that LF markedly influenced the tryptophan metabolism pathway, enriching several metabolites, including 5-Hydroxyl-L-tryptophan, Kynurenic acid, (+/−)-Tryptophan, and Tryptophol, which are known to play crucial roles in immune responses [55]. These findings provide preliminary evidence that LF contributes to maintaining intestinal homeostasis and regulating immune and metabolic activities. Furthermore, we observed that LF conferred significant protective effects against DSS-induced colitis. LF intervention markedly alleviated symptoms of weight loss, diarrhea, and rectal bleeding caused by DSS exposure and effectively prevented pathological changes such as colon shortening, increased DAI scores, and elevated spleen indices, indicating its pronounced ameliorative effect on colitis. These results are consistent with the findings of Yang et al., who reported that *Bifidobacterium breve CCFM683* alleviates colitis [56]. Histopathological analysis further demonstrated that LF significantly reduced pathological damage to colonic tissue and lowered histopathological scores, corroborating its protective effects against colitis. Collectively, these findings confirm that LF possesses anti-inflammatory potential, effectively modulates the composition of gut microbiota and microbial metabolic pathways, and alleviates the pathological phenotype of DSS-induced colitis.

Dysbiosis of the gut microbiota and oxidative stress play critical roles in the pathogenesis of intestinal diseases. Probiotics have been shown to effectively maintain intestinal health by enhancing antioxidant enzyme activity and mitigating oxidative damage [57,58]. Excessive inflammatory responses promote the overproduction of reactive oxygen species (ROS) and other free radicals, disrupting the oxidative–antioxidative balance and inhibiting the activation of the Nrf2–Keap1 signaling pathway. This results in compromised intestinal barrier function, exacerbating tissue damage and inflammation [59,60,61]. Moreover, the imbalance in oxidative stress further induces intestinal epithelial cell damage and triggers inflammatory cascades, creating a vicious cycle [62,63]. Malondialdehyde (MDA), a byproduct of lipid peroxidation, serves as a biomarker for oxidative damage and cell membrane integrity loss. Conversely, reduced activity of antioxidant enzymes reflects impairment of the body’s antioxidative defense system [64]. In this study, the DSS-treated group exhibited significantly elevated serum MDA levels alongside reduced activities of antioxidant enzymes such as T-SOD, GSH-Px, and CAT, indicating exacerbated lipid peroxidation and weakened antioxidative defense mechanisms. This led to excessive free radical accumulation and heightened oxidative stress levels. Preventive LF supplementation significantly ameliorated DSS-induced oxidative stress. Compared to the DSS group, the LF + DSS group showed reduced serum MDA levels and increased activities of T-SOD, GSH-Px, and CAT, indicating that LF effectively enhanced the body’s antioxidative defenses, mitigated lipid peroxidation damage, and preserved cell membrane integrity. Myeloperoxidase (MPO), a key marker of inflammatory damage, correlates strongly with intestinal inflammation severity [65,66]. DSS exposure significantly elevated MPO activity in colonic tissues, reflecting intensified inflammation accompanied by neutrophil infiltration and tissue damage. LF intervention markedly reduced MPO activity, demonstrating its capacity to mitigate DSS-induced inflammatory responses, reduce neutrophil infiltration, and decrease overall inflammation levels. Additionally, in the DSS group, Keap1 protein expression was significantly upregulated, whereas the expression of Nrf2 and its downstream antioxidative proteins HO-1 and NQO-1 was significantly suppressed. This suggests that DSS exposure inhibited the activation of the Nrf2–Keap1 signaling pathway, impairing the body’s antioxidative and anti-inflammatory defenses, thereby increasing susceptibility to free radical-induced damage and exacerbating tissue injury and inflammation [67]. Preventive LF supplementation significantly downregulated Keap1 protein expression and upregulated Nrf2, HO-1, and NQO-1 expression, indicating that LF regulates oxidative stress levels by activating the Nrf2–Keap1 signaling pathway. This activation enhances antioxidative capacity and suppresses inflammation. LF may influence the expression and activity of key proteins in the Nrf2–Keap1 pathway by modulating cellular signaling or intracellular environments, such as maintaining the redox balance and regulating ion concentrations, thereby strengthening antioxidative and anti-inflammatory responses [68]. In conclusion, LF enhances the body’s antioxidative and anti-inflammatory capabilities through activation of the Nrf2–Keap1 signaling pathway, alleviating DSS-induced oxidative stress and intestinal inflammatory damage.

In the study of host–microbe interactions, probiotics, particularly lactic acid bacteria, have been demonstrated to effectively modulate the host immune system and maintain intestinal health [69,70]. Lactic acid bacteria alleviate inflammatory responses by improving the intestinal immune microenvironment and promoting the secretion of immunomodulatory factors [24]. During intestinal inflammation, the balance between pro-inflammatory cytokines (e.g., IL-1β, IL-6, TNF-α, and IFN-γ) and anti-inflammatory cytokines (e.g., IL-4 and IL-10) is critical. Elevated pro-inflammatory cytokines disrupt the intestinal mucosal barrier and exacerbate inflammation, while reduced anti-inflammatory cytokines result in immune imbalance, making inflammation difficult to control and accelerating the progression of UC [71]. Notably, TNF-α induces the secretion of IL-1β and IL-6, further damaging the intestinal mucosal barrier [72]. IL-6 enhances inflammation by activating antigen-presenting cells (APCs) and T cells, and IL-1β expression levels positively correlate with the severity of inflammation [73]. Conversely, IL-10 reduces inflammation by inhibiting the expression of pro-inflammatory cytokines and chemokines through a negative feedback mechanism [74]. In this study, DSS exposure significantly elevated serum levels of pro-inflammatory cytokines (e.g., IL-1β, IL-6, TNF-α, and IFN-γ), indicating strong inflammatory activation and an increased risk of tissue damage. Simultaneously, anti-inflammatory cytokines (e.g., IL-4 and IL-10) were markedly reduced, reflecting impaired anti-inflammatory mechanisms and further exacerbating inflammation. However, LF intervention significantly mitigated these abnormalities. Pro-inflammatory cytokines were notably reduced, while anti-inflammatory cytokines were substantially increased, suggesting that preventive LF supplementation regulates immune responses, restores immune balance, and alleviates inflammation. These findings are consistent with the study by Cheng et al. [22]. In summary, LF exhibits significant immunomodulatory effects in DSS-induced UC by promoting the production of anti-inflammatory cytokines and inhibiting the secretion of pro-inflammatory cytokines. This effectively mitigates intestinal inflammation and supports intestinal health and functionality.

The intestinal mucosal barrier plays a crucial role in maintaining intestinal health, effectively preventing pathogen invasion and sustaining intestinal microbial homeostasis [75,76]. In intestinal diseases such as UC, the barrier function is compromised, with decreased expression of tight junction proteins (e.g., ZO-1, occludin, claudin-1), leading to increased intestinal epithelial permeability. This creates a pathway for external stimuli to invade, thereby exacerbating the inflammatory response [77]. In this study, DSS exposure significantly reduced the expression of tight junction proteins (e.g., ZO-1, occludin, claudin-1, and E-cadherin), as indicated by disrupted staining patterns, weakened intensity, and blurred fluorescent signals, suggesting severe impairment of intestinal barrier function. However, LF intervention significantly improved these markers, with more continuous staining of tight junction proteins and increased fluorescence intensity, indicating that LF effectively restored DSS-induced barrier damage. It is speculated that LF, through its anti-inflammatory and immunomodulatory effects, reduces the release of pro-inflammatory cytokines, creating a favorable environment for intestinal barrier repair, thereby restoring the normal expression and localization of tight junction proteins and ultimately repairing the intestinal mucosal barrier function.

The gut microbiota and their metabolites play a critical role in the pathological development of UC [78]. Studies have shown that gut dysbiosis not only directly contributes to the onset of UC but also exacerbates the condition by affecting metabolic activity and disrupting gut function and immune homeostasis [79,80]. The imbalance in the microbiota–metabolism axis is considered one of the key mechanisms underlying UC [81,82]. In this study, we found that DSS exposure led to a decrease in the relative abundance of Firmicutes and Bacteroidetes phyla, while the abundance of Proteobacteria increased. At the genus level, the abundance of potential pathogenic genera (such as *g_Escherichia-Shigella*, *g_Staphylococcus*, *g_Streptococcus*, *g_Enterococcus*, *g_Klebsiella*, and *g_Erysipelatoclostridium*) significantly increased, whereas beneficial genera (such as *g_Dubosiella*, *g_Faecalibaculum*, *g_Candidatus_saccharimonas*, *g_Odoribacter*, *g_Roseburia*, and *g_Eubacterium_xylanophilum_group*) significantly decreased. This imbalance further exacerbates gut dysbiosis and inflammation [83]. Previous studies have confirmed that the abnormal proliferation of *Shigella* is closely related to the development of UC, Crohn’s disease (CD), and severe diarrhea in children [84,85,86,87]. In addition, a study by Li et al. demonstrated that the compound MIR2911 from honeysuckle can be absorbed through the diet and directly act on gut bacteria, reducing the abundance of *Escherichia-Shigella* and improving colitis symptoms [88]. Research by Zhang et al. showed that carvacrol and thymol (CAT) alleviated DSS-induced colitis by altering the gut microbiome composition and enhancing the abundance of *g_Turicibacter* and *g_Dubosiella* bifidobacteria [89]. Notably, in the present study, LF intervention also significantly improved gut microbiome diversity and richness, effectively modulating key microbial populations and reversing the dysbiosis state. Specifically, LF intervention restored the abundance of beneficial bacterial genera (such as *g_Dubosiella*, *g_Faecalibaculum*, *g_Candidatus_saccharimonas*, *g_Coriobacteriaceae_UCG-002*, *g_Monoglobus*, *g_Odoribacter*, *g_Roseburia*, and *g_Eubacterium_xylanophilum_group*), while reducing the abundance of potential pathogenic genera (such as *g_Escherichia-Shigella*, *g_Enterococcus*, *g_Streptococcus*, *g_Staphylococcus*, *g_Klebsiella*, *g_Erysipelatoclostridium*, *g_Coprobacillus*, and *g_Corynebacterium*), thereby promoting the restoration of microbial homeostasis and alleviating inflammation [90,91].

Furthermore, metabolomic analysis revealed significant differences in the metabolite composition between treatment groups, especially distinguishing the DSS group from others. This result is consistent with Xie et al.’s study, which confirmed the significant impact of DSS on the metabolic profile [92]. LF intervention reshaped the metabolic functional pathways of the gut microbiota, involving significant changes in 312 metabolites, with tryptophan metabolism and indole and its derivatives being the most prominent. In the LF + DSS group, the levels of tryptophan metabolites (such as melatonin, kynurenic acid, 5-methoxytryptophan, and 3-indoleacetic acid) significantly increased. These metabolites possess anti-inflammatory properties and regulate intestinal immune responses to alleviate UC symptoms [93,94]. Moreover, these metabolites are closely associated with oxidative stress. Studies have shown that melatonin possesses natural antioxidant properties, improving gut health by reshaping the composition of gut microbiota, inhibiting oxidative stress, and autophagy [95]. Additionally, melatonin inhibits the production of reactive oxygen species (ROS), reduces the formation of the lipid peroxide 4-HNE [96], and increases goblet cell numbers to mitigate cadmium-induced intestinal mucosal damage [97]. Kynurenic acid (KYNA), as a reactive oxygen species (ROS) scavenger, influences oxidative stress levels in the brain structures of sheep by regulating gene expression and the activities of SOD2, CAT, and GPx1 [98]. Additionally, in a neonatal hypoxia-ischemia (HI) model, KYNA has demonstrated significant antioxidant potential and neuroprotective effects [99]. 5-methoxytryptophan (5-MTP), a natural anti-inflammatory metabolite, has also gained significant attention in recent years. Ma et al. found that 5-MTP alleviates pathological lung injury in LPS-exposed mice by reducing oxidative stress levels, upregulating the expression of Nrf2 and HO-1 proteins [100]. Consistent with Ma’s findings, Sun et al. reported that 5-MTP reduces oxidative stress and inflammation in mice with sepsis-induced acute kidney injury (S-AKI) and activates the Nrf2 signaling pathway to alleviate kidney damage post-sepsis [101]. Moreover, 5-MTP prevents cardiac injury after myocardial infarction by promoting mitochondrial stability and regulating redox imbalance [102]. KEGG pathway enrichment analysis further showed that LF significantly influenced several key metabolic pathways related to energy and nutrient metabolism, including amino acid metabolism, sphingolipid signaling pathways, and linoleic acid metabolism. These alterations were closely linked to the reshaping of the microbial community structure, particularly evident in the increase in beneficial genera. Studies have shown that *Lactobacillus* and its secondary metabolites play an important role in regulating gut immunity, maintaining barrier function, and controlling inflammation [52]. These results suggest that by modulating the composition and metabolic function of the gut microbiota, LF improves the intestinal microenvironment and effectively alleviates DSS-induced colitis. This effect is not only reflected in promoting the proliferation of beneficial bacteria and suppressing potential pathogens but also through the regulation of key metabolic pathways and enhancement of tryptophan metabolism. This study for the first time systematically reveals how LF maintains gut health and intervenes in inflammation by reshaping the community of microbiota and their metabolic network. These findings highlight the potential of targeting the microbiota and their metabolic functions in the treatment of IBD and provide new insights and theoretical support for LF as a precision microbiome therapy.

## 5. Conclusions

In summary, this study systematically demonstrates the significant protective effects of *Lactobacillus fermentum 016* (LF) in DSS-induced colitis. LF enhances the host’s antioxidant capacity by activating the Nrf2–Keap1 signaling pathway, thereby alleviating oxidative stress damage. LF also reshapes the gut microbiota, increasing microbial diversity and the abundance of beneficial bacteria, while inhibiting the expansion of potential pathogenic bacteria. Additionally, LF enhances tryptophan metabolism, producing key metabolites that have anti-inflammatory and tissue-protective effects, thereby effectively improving colonic inflammation. This study is the first to elucidate the molecular mechanisms by which LF mitigates intestinal inflammation through the regulation of the microbiota–metabolism axis. It highlights the potential of targeting the gut microbiota in the treatment of IBD and provides new insights and theoretical support for the application of probiotics in IBD therapy.

## Data Availability

The raw sequencing data have been deposited into the National Center for Biotechnology Information (NCBl) Sequence Read Archive (SRA) database under accession numbers PRJNA1213313, and PRJNA1213299. All relevant data are available from the authors.
